# Deletion of the s2m RNA Structure in the Avian Coronavirus Infectious Bronchitis Virus and Human Astrovirus Results in Sequence Insertions

**DOI:** 10.1128/jvi.00038-23

**Published:** 2023-02-13

**Authors:** Sarah Keep, Giulia Dowgier, Valeria Lulla, Paul Britton, Michael Oade, Graham Freimanis, Chandana Tennakoon, Christine Monceyron Jonassen, Torstein Tengs, Erica Bickerton

**Affiliations:** a The Pirbright Institute, Woking, United Kingdom; b The Francis Crick Institute, London, United Kingdom; c Department of Pathology, University of Cambridge, Addenbrookes Hospital, Cambridge, United Kingdom; d Lewis Thomas Laboratory, Department of Molecular Biology, Princeton University, Princeton, New Jersey, USA; e Department of Virology, Norwegian Institute of Public Health, Oslo, Norway; f Department of Breeding and Genetics, Nofima, Ås, Norway

**Keywords:** infectious bronchitis virus, coronavirus, human astrovirus, s2m, RNA structure

## Abstract

Coronaviruses infect a wide variety of host species, resulting in a range of diseases in both humans and animals. The coronavirus genome consists of a large positive-sense single-stranded molecule of RNA containing many RNA structures. One structure, denoted s2m and consisting of 41 nucleotides, is located within the 3′ untranslated region (3′ UTR) and is shared between some coronavirus species, including infectious bronchitis virus (IBV), severe acute respiratory syndrome coronavirus (SARS-CoV), and SARS-CoV-2, as well as other pathogens, including human astrovirus. Using a reverse genetic system to generate recombinant viruses, we investigated the requirement of the s2m structure in the replication of IBV, a globally distributed economically important *Gammacoronavirus* that infects poultry causing respiratory disease. Deletion of three nucleotides predicted to destabilize the canonical structure of the s2m or the deletion of the nucleotides corresponding to s2m impacted viral replication *in vitro*. *In vitro* passaging of the recombinant IBV with the s2m sequence deleted resulted in a 36-nucleotide insertion in place of the deletion, which was identified to be composed of a duplication of flanking sequences. A similar result was observed following serial passage of human astrovirus with a deleted s2m sequence. RNA modeling indicated that deletion of the nucleotides corresponding to the s2m impacted other RNA structures present in the IBV 3′ UTR. Our results indicated for both IBV and human astrovirus a preference for nucleotide occupation in the genome location corresponding to the s2m, which is independent of the specific s2m sequence.

*Orthocoronaviridae* is a subfamily of viruses, commonly referred to as coronaviruses, that are known to infect a variety of hosts, causing a wide variety of diseases in both humans and animals. These viruses are divided into one of four genera, *Alphacoronavirus*, *Betacoronavirus*, *Gammacoronavirus*, and *Deltacoronavirus*. The avian coronavirus infectious bronchitis virus (IBV) is a member of the *Gammacoronavirus* genus that affects domestic fowl (1, 2), primarily infecting the host respiratory tract, and is a global endemic virus. Although IBV is controlled by vaccination, it remains of significant economic importance to poultry industries worldwide, as infection results in a highly contagious respiratory disease, infectious bronchitis, that results in poor weight gain, mortality, and reduced egg production (3). There are many cocirculating IBV strains of different serotypes and genotypes that inflict different and various degrees of clinical disease (2–4).

As with all coronaviruses, the genome of IBV is a large positive-sense single-stranded RNA molecule which possesses a methylated cap structure at the 5′ end and is polyadenylated at the 3′ end (5). Genome organization is shared within the *Coronaviridae* family: a large replicase gene composed of two open reading frames, ORF1a and ORF1b, which produce two poly proteins, pp1a and pp1ab, with the latter the result of a – 1 programmed ribosomal frameshift event (6, 7), followed by the structural genes spike (S), envelope (E), membrane (M), and nucleocapsid (N) (8). Interspersed among the structural genes are the accessory genes (8, 9). Flanking each end of the genome are the untranslated regions (UTR), denoted 5′ UTR and 3′ UTR. Both these regions are known to contain highly organized RNA secondary structures that are thought to play a role in genome replication as well as in the synthesis of the subgenomic mRNAs (sgmRNAs) that encode the structural and accessory proteins (10–17).

The 3′ UTR of the coronavirus genome, including that of IBV, contains several secondary and tertiary RNA structures (18), including a stem-loop (11, 15) and a pseudoknot (13, 19, 20). Both these structures have been demonstrated to play a role in coronavirus replication (13, 19–21). Additionally, a nucleotide genetic element, denoted s2m, with a highly conserved secondary (as well as primary and tertiary) RNA structure was initially identified in the IBV genome (22). The s2m has subsequently been identified in all members of the *Igacovirus* subgenus of the gammacoronaviruses, members of the *Buldecovirus* subgenus of the deltacoronaviruses, and the *Sarbecovirus* subgenus of the betacoronaviruses, including severe acute respiratory syndrome coronaviruses (SARS-CoV), and more recently, in SARS-CoV-2 (23). Interestingly, not all members of the *Orthocoronaviridae* contain the s2m structure (24); notable exceptions are members of the alphacoronaviruses (24) and most members of the betacoronaviruses, including murine hepatitis virus (MHV, subgenus *Embecovirus*) (20) and Middle Eastern respiratory coronavirus (MERS-CoV, subgenus *Merbecovirus*) (24). Additionally, the s2m motif has also been identified in astroviruses (22) and in members of the *Caliciviridae* and *Picornaviridae* (25). Due to the number of diverse virus families containing the element, an ancestral origin is considered unlikely, and it is instead proposed that the s2m sequence has been horizontally transferred via recombination during rare coinfections of the same cell by different viruses (22).

Although the crystal structure of the s2m structure has been solved in the context of SARS-CoV (26), the function in coronavirus replication remains elusive, with several hypotheses reported, including the hijacking of host protein synthesis (26) and RNA interference (RNAi)-based gene regulation (24, 27). One report considered that due to the likelihood that s2m has transferred between unrelated viruses, the target for the s2m function is host specific (24). Although both the function and significance remain unknown, the conserved nature of the s2m structure has raised the possibility of it being an antiviral drug target (26) as well as a target for virus discovery assays (28). The identification of s2m in the recently emerged SARS-CoV-2 (23, 29) has led to further interest in its role and its potential as an antiviral drug target (29).

The aim of this study was to determine whether s2m is required for coronavirus replication using the model *Gammacoronavirus* IBV. Two recombinant IBVs (rIBVs) were generated using a vaccinia virus-based reverse genetics system based on the apathogenic laboratory Beaudette strain (30, 31). Three nucleotides (GCC) were deleted, disrupting the canonical stem-loop structure, and the 41 nucleotides encoding the entire s2m sequence were removed, generating the rIBVs BeauR-delGCC and BeauR-del-s2m. Replication of both rIBVs in *vitro* was found to be comparable to the parental Beau-R but was reduced in tracheal organ cultures, an *ex vivo* model for *in vivo* IBV infection. Interestingly, *in vitro* passage of BeauR-dels2m identified a 36-nucleotide insertion in place of the deleted s2m sequence. This insertion did not consist of a s2m-like sequence but, rather, consisted of duplicated nucleotide sequences located both upstream and downstream of the original s2m sequence. *In vitro* passaging of a human astrovirus (HAstV1) also containing a deletion in the s2m identified a similar duplication. Our results therefore indicate that there is a preference for both IBV and HAstV1 to retain the nucleotide sequence in place of the s2m and, additionally, that the canonical structure of the s2m may play a role during *in vivo* infection.

## Results

### Generation of rIBVs either with a disrupted canonical s2m stem-loop structure or completely lacking the s2m sequence

**FIG 1:**
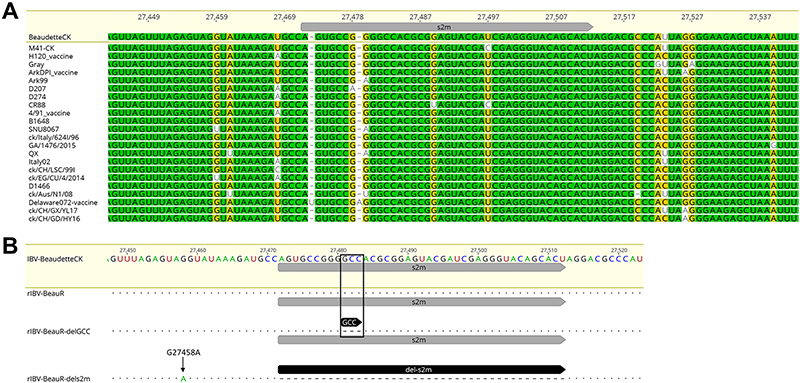
Schematic detailing the modifications to s2m in BeauR-delGCC and BeauR-dels2m. (A) Sequence alignment of the s2m region in IBV strains belonging to different genotypes and serotypes. The MAFTT alignment was generated using Geneious 10.2.3 with percentages of identity (%) displayed by colors (100%, green; 80 to 100%, mustard yellow; 60 to 80%, yellow; <60%, white). The strains and accession numbers are as follows: Beau-CK (AJ311317), M41-CK (MK728875.1), H120 (MN548287), Gray (GU393334.1), ArkDPI11_vaccine (EU418976), Ark99 (MH779860), D207 (AJ278335), D274 (MH021175), CR88 (MN548285.2), 4/91_vaccine (KF377577), B1648 (KR231009), SNU8067 (JQ977697), ck/Italy/624I/96 (MG021194), GA/1476/2015 (MN599049), QX (MN548289), Italy-02 (MN548288), ck/CH/LSC/99I (KY799582), ck/EG/CU/4/2014 (KY805846), D1466 (MN548286), ck/Aus/N1/08 (KU556807), Delaware072_vaccine (GU393332), ck/CH/GX/YL17 (MK329221), ck/CH/GD/HY16 (MK309398). (B) Genome alignment highlighting the positions of the modifications to the 3′ UTR. Nucleotides GCC, highlighted by the black box, were deleted generating BeauR-delGCC, and the 41 nucleotides encoding the s2m were deleted generating BeauR-dels2m. The G to A nucleotide mutation detected in BeauR-dels2m at position 27458 is marked by the arrow. MAFTT alignment was generated using Geneious 10.2.3 using the Beau-CK reference sequence (accession number AJ311317).

Comparison of s2m sequences from several strains of IBV representing diverse serotypes as well as the genotypes defined by Valastro et al. (4) demonstrated that the sequence is largely conserved (Fig. 1A). Two strains were identified that had additional nucleotides and two that contained single point mutations. The laboratory strain Beau-CK (accession number AJ311317) was chosen as a representative strain.

To determine whether the s2m sequence and the associated stem-loop structure are required for the replication of IBV, two rIBVs, BeauR-delGCC and BeauR-dels2m, were generated using a vaccinia virus-based reverse genetic system previously established for the apathogenic IBV strain Beau-CK (30). The first rIBV, BeauR-delGCC, has the three nucleotides, GCC, at positions 27481 to 27483 deleted (Fig. 1B). The loss of these three nucleotides is predicted to disrupt the canonical hairpin stem-loop of the s2m structure. The three nucleotides are among the most conserved residues in viruses harboring s2m across different viral families. The second rIBV, BeauR-dels2m, has nucleotides representing positions 27472 to 27512 deleted, accounting for the entire s2m nucleotide sequence (Fig. 1B). Both rIBVs were recovered from the corresponding recombinant vaccinia virus vectors in chicken kidney (CK) cells with stock viruses generated after 3 passages in CK cells and 1 in embryonated hen's eggs (31).

The recovery of a rIBV from its vaccinia is typically carried out in replicates of 10 to increase the probability of successful rescue (31). For BeauR-delGCC, all 10 replicates were positive for the recovery of the rIBV, largely comparable to the control parent virus, Beau-R, in which 8 out of 10 replicates were positive. Sequencing of the 3′ UTR of each of the recovered rIBVs confirmed that they all contained the GCC deletion, and therefore one replicate was taken forward for generation of stock virus for use in all subsequent experiments. The recovery of rIBV BeauR-dels2m, however, was not as straightforward. The first attempt at rescue of rIBV BeauR-dels2m in CK cells, alongside that of BeauR-delGCC, yielded no recombinant virus (0/10 replicates). For the second attempt, two recombinant vaccinia virus clones, identified to contain the correct modified IBV 3′ UTR sequence, were taken forward. Only one replicate from one rVV clone yielded infectious rIBV in comparison to 10 of 10 replicates for the control, Beau-R. This meant the overall success rate for the rescue of rIBV BeauR-dels2m was 1 out of 30 (3.3%). Additionally, sequence analysis identified that the only rescued isolate also contained a point mutation, G27458A, located 13 nucleotides upstream of the deleted sequence. This mutation was not present in the BeauR-dels2m IBV cDNA sequence within the vaccinia vector, indicating that it occurred during the recovery process, a finding that raised the possibility that this mutation may play a compensatory role.

### Changes to the s2m sequence did not result in compensatory mutations within the 5′ UTR

The 3′ UTR, in which the s2m is located, physically interacts with the 5′ UTR during coronavirus replication, specifically stimulating subgenomic mRNA synthesis (32, 33). The 5′ UTR sequences of the stock viruses representing both rIBVs, BeauR-delGCC and BeauR-dels2m, were sequenced alongside the parental virus, Beau-R. No nucleotide sequence changes were identified in the 5′ UTRs of either rIBV, indicating that either the s2m motif does not interact directly with the 59 UTR or the changes present in the s2m motif of rIBVs BeauR-delGCC or BeauR-dels2m had not impacted any potential interaction with the 5′ UTR requiring a compensatory mutation.

### A 36-nucleotide insertion occurs in the 3′ UTR of BeauR-dels2m following in vitro passage

**FIG 2:**
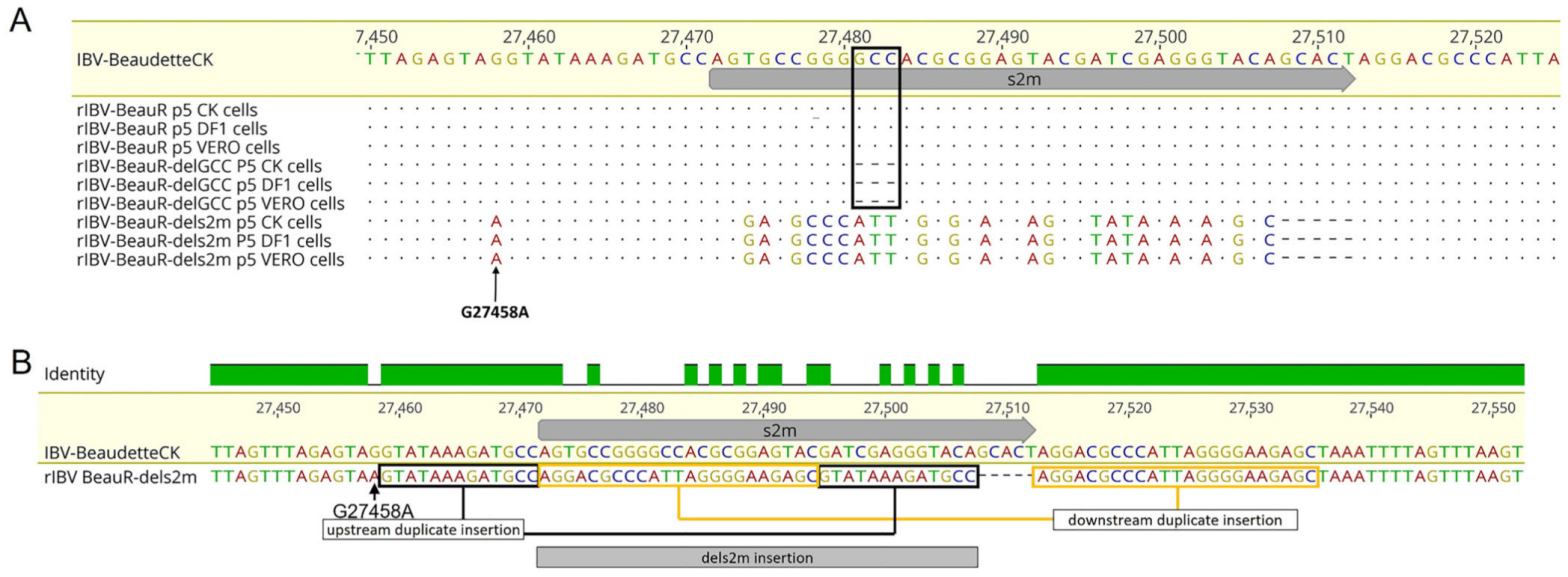
*In vitro* passaging of BeauR-dels2m has resulted in a 36-nt insertion in place of the deleted s2m sequence. (A) BeauR-delGCC and BeauR-dels2m were passaged in CK, Vero, and DF1 cells in replicates of three. The 3′ UTRs of the resulting viruses were Sanger sequenced at passage five. Reads from the sequencing results were mapped to the reference sequence of IBV Beaudette-CK (accession number AJ311317) using Geneious 10.2.3. The results of the replicates were identical. No additional mutations were observed in sequences obtained from Beau-R or BeauR-delGCC in any of the cell types. The black rectangle identifies the location of the GCC deletion. A 36-nt insertion was detected in passaged BeauR-dels2m isolates, with the insertion identified in all cell types. The G27458A mutation detected in the viral stock was retained and is marked by the arrow. (B) The 36-nt insertion identified in BeauR-dels2m consists of duplicate sequences that are located up- and downstream of the deleted s2m sequence.

The rIBVs BeauR-delGCC and BeauR-dels2m were serially passaged five times in triplicate in CK, Vero, and DF1 cells alongside parental Beau-R. RNA was extracted from the cell culture supernatants of the passage 5 viruses and assessed for the presence of IBV RNA using reverse transcriptase PCR (RT-PCR) targeting the 3′ UTR. In all replicates in all cell lines, a single PCR product of ∼650 bp was observed, representing the region of the IBV 3′ UTR containing the s2m sequence. The PCR products were sequenced (Fig. 2A), and no mutations were identified in either the Beau-R or BeauR-delGCC 3′ UTR (Fig. 2A). In contrast to both Beau-R and rIBV BeauR-delGCC, the sequence of the 3′ UTR of BeauR-dels2m revealed an insertion of 36 nucleotides in place of the deleted s2m sequence (Fig. 2A). Interestingly, the initial nucleotide of the 36-nucleotide (nt) insertion was not only at the exact position of the first nucleotide of the deleted s2m sequence, but it was also an adenosine residue, the same as the corresponding nucleotide of the IBV s2m motif. The inserted sequence was five nucleotides shorter than the s2m sequence and did not show any homology to the s2m sequence.

Analysis of the sequence revealed that the nucleotides inserted were sequential duplicates of sequences already present within the IBV Beaudette 3′ UTR, nucleotides bordering the site of the s2m deletion (Fig. 2B). The G27458A mutation is retained and marked the start of the duplicated sequence upstream from the s2m deletion site. Further investigation identified the 36-nt insertion in all the intermediate passages. This suggested either that this insertion occurred rapidly from the point at which the stock virus was generated or that the stock virus contained the insertion at a level that was not detectable via PCR and Sanger sequencing. Additionally, as part of the analysis of the passaged viruses, we also sequenced the 5′ UTR; no changes were identified.

### Low-level detection of the 36-nt insertion in the rIBV BeauR-dels2m stock virus

Although Sanger sequencing of the stock virus of BeauR-dels2m did not detect the 36-nt insertion, as indicated above it was possible that a viral genome containing the insertion was present as a minority population and that subsequent growth of the rIBV resulted in the virus with the insert becoming dominant. To test this possibility, the stock virus of rIBV BeauR-dels2m and one isolate of the *in vitro*-passaged rIBV BeauR-dels2m (BeauR-dels2m-P1-CK) were investigated using next-generation sequencing (NGS). The 36-nt insertion was identified in both. To estimate the proportion of the population with the 36-nt insertion, the longest contigs that either contained or did not contain the sequence GTATAAAGATG CCAGGACGCCCATTAGGGGAAGAGC, accounting for the majority of the 36-nt insertion plus 10 nucleotides upstream, were extracted from the data set. Reads were then mapped to the combination of these genomes with the Burrows-Wheeler MEM aligner (BWA-MEM) (34) with the setting to assign one hit per read (-a option). This gave a reasonable, though not exact, assignment of reads to the region where the 36-nt insertion would appear (Table 1). The average read depth of the region flanking 10 nucleotides at the start of this insertion was calculated. These average depths were taken to be the count of reads for the variant position when calculating the allele frequency of the 36-nt insertion. While this is not exact, it gave a rough idea of these allele proportions and showed that the presence of the 36-nt insertion was greater in the passaged virus than in the stock virus. This taken together with the lack of detection of the 36-nt insertion in the stock virus but detection in the subsequent passaged viruses by Sanger sequencing suggests that the 36-nt insertion was present in the stock of rIBV BeauR-dels2m as a minority population. This population then became dominant through further passages, suggesting that the presence of the 36-nt insertion has some intrinsic advantage for viral replication.

Overall, the difficulty in recovering an isolate of BeauR-dels2m would suggest that s2m is required for viral replication, However, despite the stock virus containing the 36-nt insertion as a minority population, it does not contain the s2m sequence; this has been successfully deleted. Therefore, the successful recovery of a virus lacking the s2m sequence followed by the subsequent insertion of 36 nt indicates that although the nucleotides encoding the s2m sequence are not essential for replication *in vitro*, it appears there is a requirement to have a nucleotide sequence occupying the s2m position in the genome. The successful recovery of BeauR-delGCC and subsequent passaging with no additional mutations also suggests that the canonical hairpin structure of the s2m is also not required for viral replication. Additionally, there does not appear the need for any compensatory mutations within the 5′ UTR following the loss of either the s2m structure or s2m nucleotides.

### The insertion of 36 nucleotides in BeauR-dels2m alters the predicted RNA structure of the 3′ UTR

Both the NGS data and in *vitro* passaging (Fig. 2) indicated that a minority population of the rescued rIBV BeauR-dels2m contained a 36-nt insertion that became the dominant population on further passaging in three different cell types. As a result, the stock virus cannot be considered a ″clean″ total s2m deletion mutant, and therefore, the stock of this virus which is used in all subsequent experiments is denoted from this point forward as BeauR-dels2m-36nt-INS.

**FIG 3:**
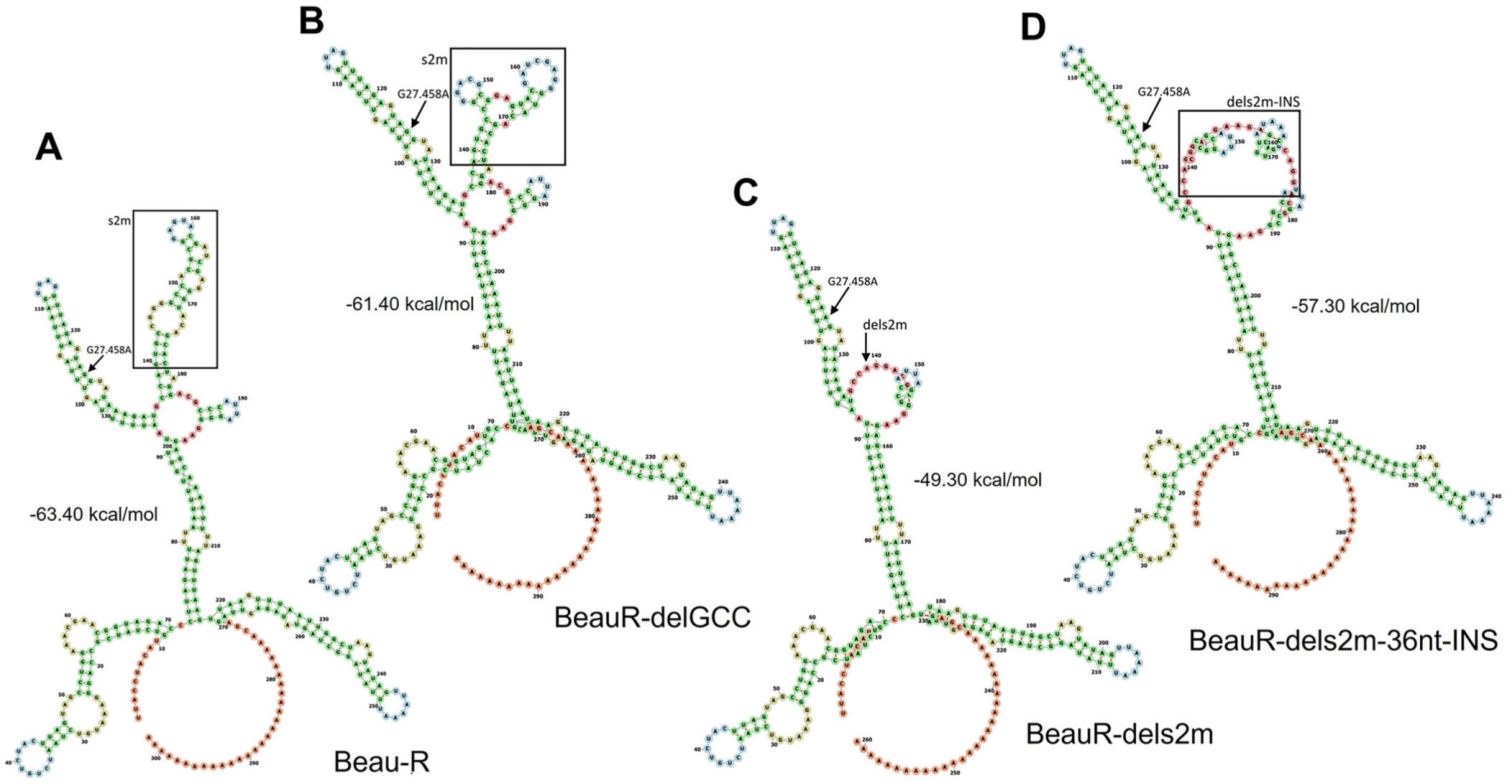
The deletion of the nucleotides corresponding to s2m is predicted to affect the structure of the 3′ UTR. (A to D) The predicted RNA secondary structure of (A) Beau-R, (B) BeauR-delGCC, (C) BeauR-dels2m, and (D) BeauR-dels2m containing the 36-nucleotide insertion, denoted BeauR-dels2m-36nt-INS. The secondary structure prediction was performed using the RNAfold WebServer based on ViennaRNA Package 2.0. Nucleotides are numbered starting at position 27,334 (Beaudette-CK; https://www.ncbi.nlm.nih.gov/nuccore/AJ311317), and similar colors represent similar predicted structures. The G27458 mutation is shown by the arrow, and the s2m is framed by a square. The sequence of the 3′ UTR of Beau-R is identical to that of Beau-CK.

RNA modeling using the RNAfold WebServer was used to investigate whether the 36-nt insertion had potentially resulted in the insertion of a new compensatory RNA structure to replace the s2m stem-loop. The secondary RNA structures present within the 3′ UTR of Beau-R and rIBVs BeauR-delGCC, BeauR-dels2m, and BeauR-dels2m-36nt-INS were compared (Fig. 3). The 3′ UTR of IBV was previously considered to start immediately after the N protein stop codon and was divided into two regions—the hypervariable and conserved regions (9). Research has identified that the hypervariable region encodes a previously unrecognized ORF denoted 7, nucleotide position 27111 to 27333, which is deleted in the M41 strain (35, 36). We therefore modeled the nucleotide sequence of the 3′ UTR after the stop codon of this ORF, as it is this sequence that is the most conserved among IBV strains.

The predicted secondary structure of the 3′ UTR of Beau-R (Fig. 3A) is highly complex, containing several distinct structures (9, 15). The predicted structure was found to have high stability (−63.40 kcal/mol minimum free energy). The projection of the Beau-R 3′ UTR with the G27458A mutation did not alter any of the predicted structures but did improve the stability by decreasing the free energy value by −0.4 kcal/mol to −63.80 kcal/mol. Modeling of the BeauR-delGCC 3′ UTR (Fig. 3B) resulted in an altered conformation of the s2m structure but did not affect the other IBV 3′ UTR predicted structures. The increase in the minimum free energy (−61.40 kcal/mol) suggests that the 3′ UTR of BeauR-delGCC is slightly less stable than that of Beau-R.

The predicted 3′ UTR structure following the 41-nucleotide deletion encoding the s2m structure in BeauR-dels2m, is altered in comparison to that of Beau-R. Not only is the s2m sequence removed, but also, the adjacent hairpin loop structure is affected (Fig. 3C). The calculated free energy value is −49.30 kcal/mol, suggesting much lower stability. Interestingly, the predicted stability of the structure improves (−57.30 kcal/mol) when the 36-nt insertion is included (Fig. 3D) for the 3′ UTR of rIBV BeauR-dels2m-36nt-INS. However, the insertion is not predicted to restore either an s2m-like stem-loop or the adjacent hairpin loop. Despite this, there is clearly a change in the overall predicted structure of the BeauR-dels2m-36nt-INS 3′ UTR.

**FIG 4:**
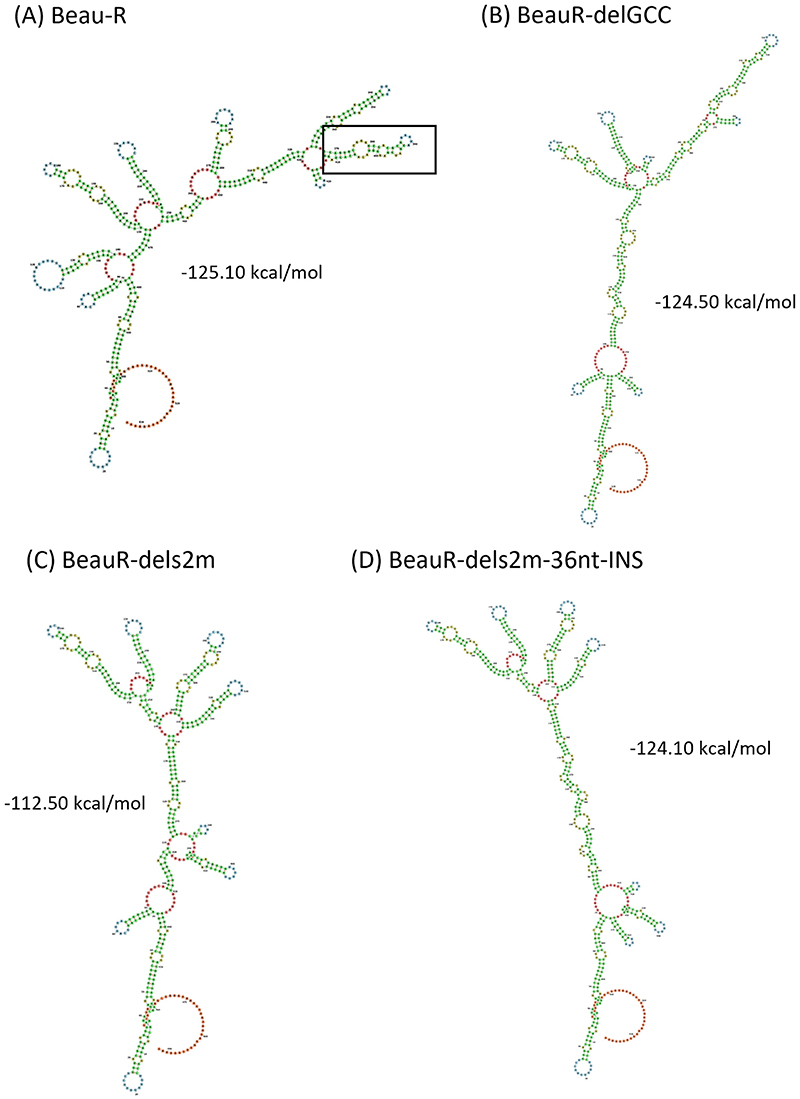
The deletion of the nucleotides corresponding to s2m is predicted to affect the structure of gene 7 and the 3′ UTR. (A to D) The predicted RNA secondary structure of (A) Beau-R, (B) BeauR-delGCC, (C) BeauR-dels2m, and (D) BeauR-dels2m containing the 36-nucleotide insertion, denoted BeauR-dels2m-36nt-INS. The secondary structure prediction was performed using the RNAfold WebServer based on ViennaRNA Package 2.0. Nucleotides are numbered starting at position 27,103 (Beaudette-CK https://www.ncbi.nlm.nih.gov/nuccore/AJ311317), and similar colors represent similar predicted structures. The s2m structure in panel A is framed by a square. The sequence of the 3′ UTR of Beau-R is identical to that of Beau-CK.

To investigate further and to include any potential regulatory structures that may overlap with ORF 7, we modeled the nucleotides immediately after the N stop codon to the end of the poly(A) tail (Fig. 4). The results of the modeling are different from that shown in Fig. 3 but similarly suggest that the deletion of the s2m has altered the RNA structures present. The deletion of the complete s2m sequence has also changed the minimum free energy, −112.50 kcal/mol in comparison to Beau-R at −125.10 kcal/mol. Interestingly, the predicted minimum free energy for BeauR-dels2m-36nt-INS is −124.10 kcal/mol, comparable to both Beau-R and BeauR-delGCC (−124.50 kcal/mol).

### Disruption of the canonical stem-loop structure of s2m resulted in reduced plaque size

**FIG 5:**
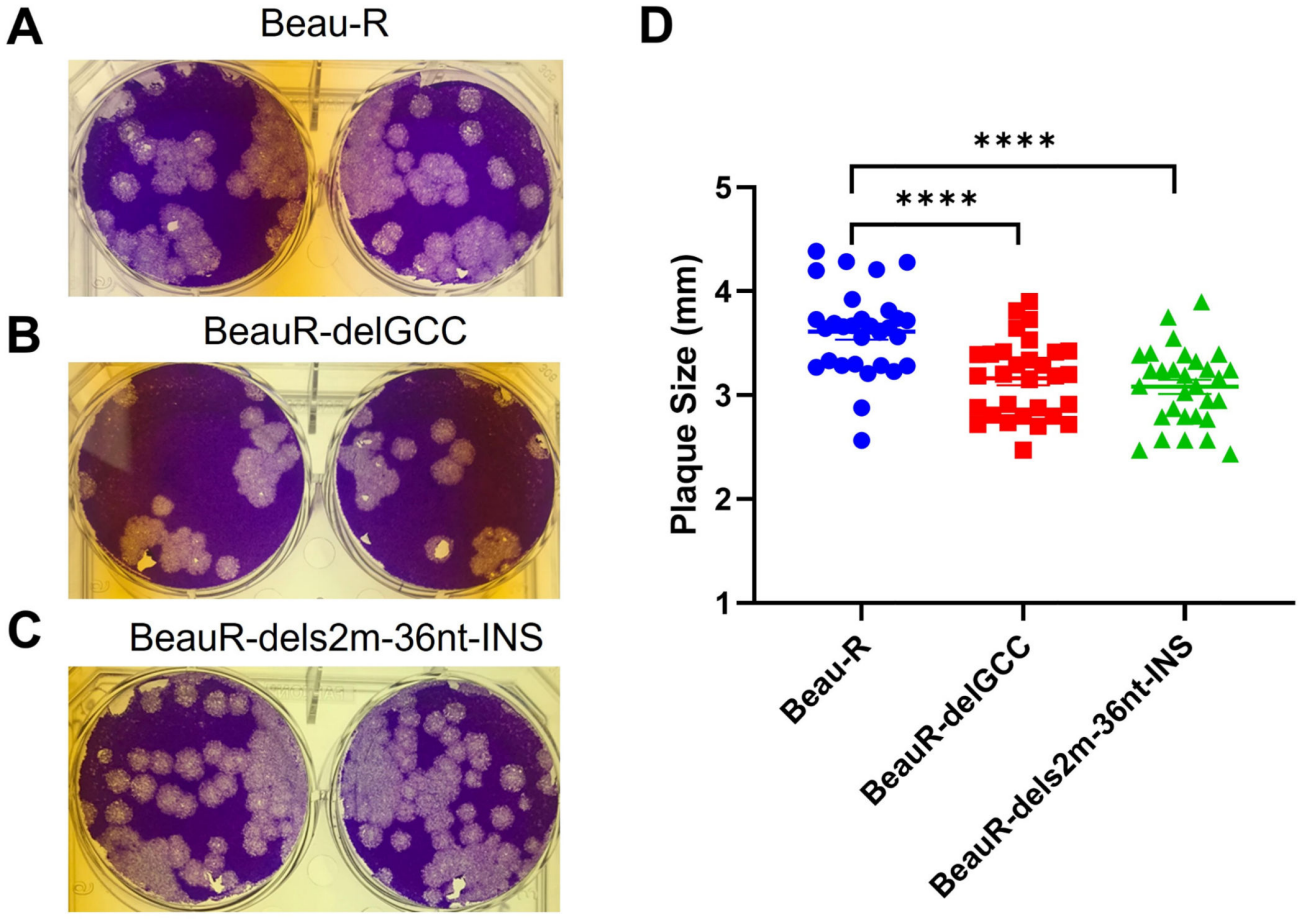
The deletion of the s2m stem-loop has resulted in reduced plaque size. (A to C) Photographs showing the plaque phenotype in CK cells for (A) Beau-R, (B) BeauR-delGCC, and (C) BeauR-dels2m-36nt-INS. Infected CK cells were stained with 0.1% crystal violet 4 days postinfection. (D) The diameter of 30 individual plaques in CK cells was measured using ImageJ. Statistical differences were analyzed using a one-way analysis of variance (ANOVA) with a Tukey test for multiple comparisons and are highlighted by asterisks: ****, *P* < 0.0001.

Comparable plaque morphology in CK cells was observed between both rIBVs, BeauR-delGCC and BeauR-dels2m-36nt-INS, and the parental Beau-R (Fig. 5A to C). However, the plaque diameters of the two rIBVs were slightly reduced in comparison to Beau-R, although the plaque diameters were comparable between BeauR-delGCC and BeauR-dels2m-36nt-INS (Fig. 5D). The reduction in plaque size for both BeauR-delGCC and BeauR-dels2m-36nt-INS suggests that the loss of the canonical hairpin stem-loop structure of s2m may have a minor effect on viral replication *in vitro*.

### The s2m stem-loop is not required for replication *in vitro*

**FIG 6:**
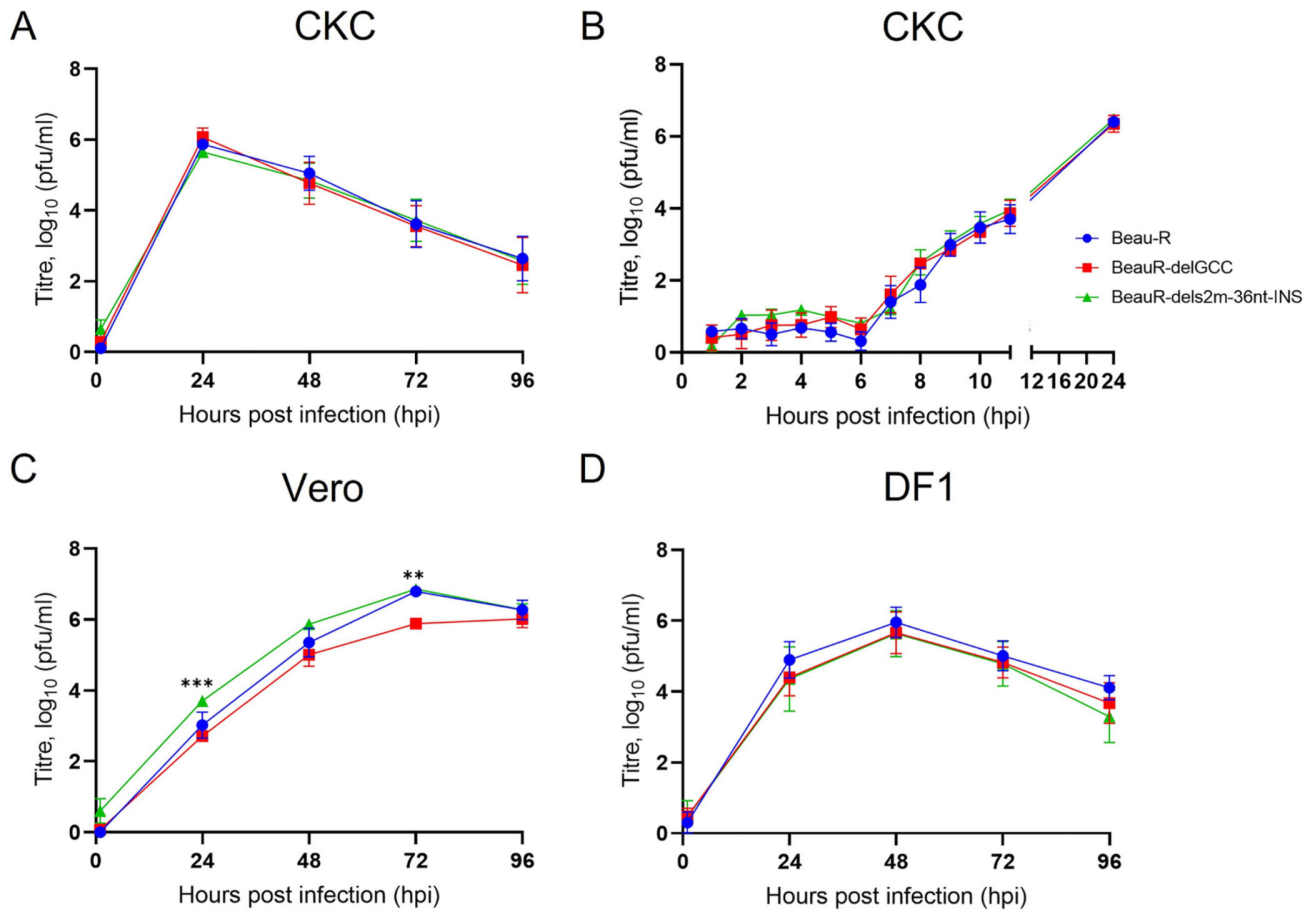
Deletion or disruption of the s2m stem-loop yields a minor replication defect in Vero cells, but not DF-1 or primary CK cells. (A to D) Primary CK cells (A and B), Vero cells (C), and DF1 cells (D) seeded in 6-well plates were inoculated with 10^4^ PFU of either Beau-R, BeauR-delGCC, or BeauR-dels2m-36nt-INS. Supernatants were harvested at 24-h intervals, and the quantity of infectious progeny was determined via titration in CK cells. Each point represents the mean of three independent experiments, with error bars representing the standard error of the mean (SEM). Statistical differences were assessed using a two-way ANOVA followed by a Tukey test for multiple comparisons. Statistical significances were only identified in Vero cells (panel C) and are highlighted by asterisks: **, *P* < 0.005 between BeauR-delGCC and BeauRdels2m-36nt-INS at 24 hpi;***, *P* < 0.0005 between both Beau-R and BeauR-delGCC as well as BeauR-delGCC and BeauR-dels2m-36nt-INS at 72 hpi.

To establish whether the disruption of the s2m stem-loop structure or the loss of the s2m nucleotide sequence impacted virus replication in *vitro*, a growth kinetic assay was carried out in primary CK cells (Fig. 6A). The titers of both rIBVs, BeauR-delGCC and BeauR-dels2m-36nt-INS, at all the time points assessed, were comparable to each other and to parental Beau-R (Fig. 6A). To rule out the possibility that the s2m stem-loop plays a role in early infection, the growth kinetic assay was repeated assessing earlier time points, as the IBV replication cycle is considered between 6 and 8 h (37); no statistical differences were observed at the earlier time points (Fig. 6B).

Beau-R exhibits extended cell tropism allowing propagation in several cell lines (38), and because the s2m stem-loop structure has been implicated in a host response (24), growth kinetic assays were carried out in Vero cells, a continuous mammalian cell line, and in DF1 cells, a continuous avian cell line (Fig. 6C to D). Vero cells are notably deficient in the type I IFN response and do not secrete either interferon (IFN) alpha or IFN beta (39). Interestingly, SARS-CoV-2 passaged on Vero E6 cells resulted in a deletion within the s2m sequence (40). Similarly, although not deficient, DF1 cells also have a weakened IFN I response (41). The growth kinetics of BeauR-delGCC and BeauR-dels2m-36nt-INS in Vero (Fig. 6C) and DF1 (Fig. 6D) cells were broadly comparable to that of parental Beau-R, with peak titers achieved in the different cells at 72 and 48 h postinfection (hpi), respectively. The results indicated that neither the loss of the s2m sequence nor disruption of the stem-loop structure had any observable effect on the growth kinetics. These observations imply that neither the canonical s2m stem-loop structure nor the exact nucleotides that constitute the s2m structure are required for IBV replication *in vitro*.

### The absence of the canonical s2m stem-loop structure or the nucleotides encoding the s2m stem-loop did not affect ciliary activity in ex *vivo* TOCs

**Figure 7:**
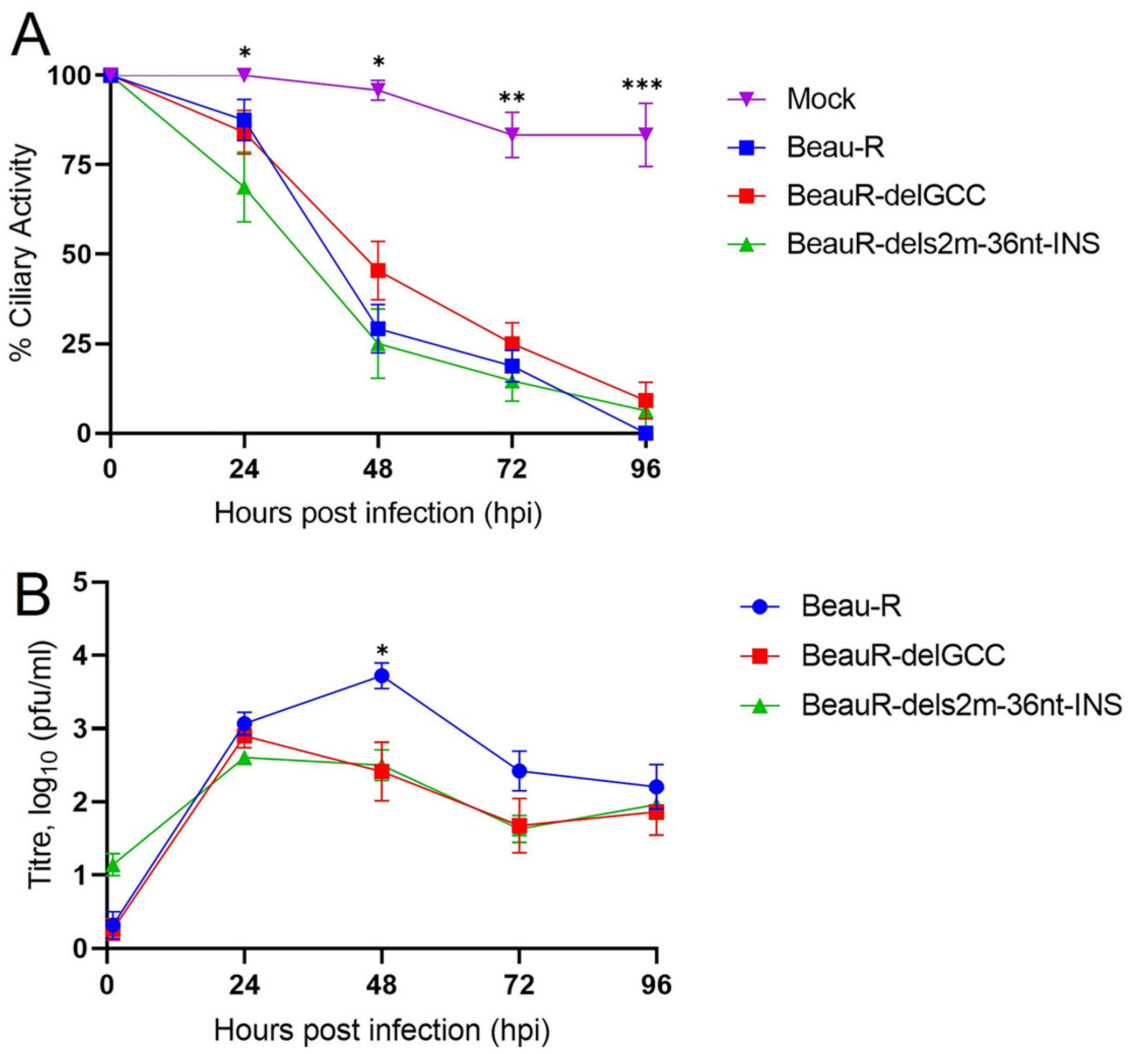
The deletion of the s2m stem-loop has reduced viral replication in *ex vivo* TOCs. (A) Replicates of 12 ex vivo TOCs, prepared from 19-day-old SPF embryos, were inoculated in with 10^4^ PFU of either Beau-R, BeauR-delGCC, BeauR-dels2m-36nt-INS, or medium for mock infection. The ciliary activity of each TOC was assessed by light microscopy at 12-h intervals, with the mean value calculated. Error bars represent the SEM. Statistical differences at each time point were analyzed using a Kruskal-Wallis test followed by Dunn's analysis for multiple comparisons. Statistical differences between mock and BeauR-dels2m-36nt-INS (24 hpi) and between mock and all rIBVs (48 to 96 hpi) are highlighted by asterisks: *, *P* < 0.05;**, *P* < 0.005;***, *P* < 0.0005. No statistical differences were highlighted between the rIBVs. (B) *Ex vivo* TOCs prepared from SPF RIR chickens were inoculated in duplicate with 10^4^ PFU of either Beau-R, BeauR-delGCC, or BeauR-dels2m-36nt-INS. Supernatants were harvested at 24-h intervals, and the quantity of infectious progeny was determined via titration in CK cells. Each point represents the mean of three independent experiments, with error bars representing the SEM. Statistical differences were assessed using a two-way ANOVA with a Tukey test for multiple comparisons. The only differences identified, between Beau-R and BeauR-delGCC, and Beau-R and BeauR-dels2m-36nt-INS are highlighted an asterisk: *, *P* < 0.05.

During *in vivo* infection, IBV replication primarily occurs in tracheal epithelial cells. Our results had indicated that neither the s2m stem-loop nor the nucleotides encoding the structure were required for replication *in vitro*. Therefore, replication of rIBVs BeauR-delGCC and BeauR-dels2m-36nt-INS were investigated in *ex vivo* tracheal organ cultures (TOCs), which are more representative of a natural infection (Fig. 7). To determine whether the two rIBVs had any impact on the ability of IBV to cause ciliostasis, the complete cessation of movement of the cilia on tracheal epithelial cells, a ciliary activity assay was performed (Fig. 7A). The cessation of tracheal cell ciliary activity is a well-used marker for *in vivo* IBV replication (42, 43). Both rIBVs caused the loss of ciliary activity, as observed for Beau-R, compared to mock-infected TOCs, indicating that the loss of the s2m stem-loop structure or the absence of the nucleotides encoding the structure did not appear to affect the ability to cause a loss of ciliary activity. Although it appeared that BeauR-dels2m-36nt-INS resulted in slightly greater reduction in ciliary activity at 24 hpi than either Beau-R or BeauR-delGCC, no statistical differences were observed between the three viruses at any time point.

### Absence of the canonical s2m stem-loop structure has reduced viral replication in *ex vivo* TOCs

To further investigate viral growth in tracheal epithelial cells, a growth kinetic assay was performed (Fig. 7B). The amount of infectious progeny virus generated from Beau-R infection peaked at 48 hpi, whereas the peak titers, observed for both BeauR-delGCC and BeauR-dels2m-36nt-INS infections, occurred earlier at 24 hpi. However, at 48 hpi the observed titers for both BeauR-delGCC and BeauR-dels2m-36nt-INS were approximately 1 log lower than the observed titer from Beau-R (*P* < 0.05), indicating some impairment in growth by the two rIBVs compared to Beau-R. BeauR-delGCC and BeauR-dels2m-36nt-INS exhibited similar growth kinetics, suggesting that it is the disruption of the canonical hairpin stem-loop structure rather than the deletion of the specific nucleotides encoding the s2m structure that has resulted in an observed altered growth phenotype in *ex vivo* TOCs.

### Passaging of human astrovirus 1 (HAstV1) lacking s2m also results in an insertion resulting from duplication of the 3′ region preceding the poly-A tail

**FIG 8:**
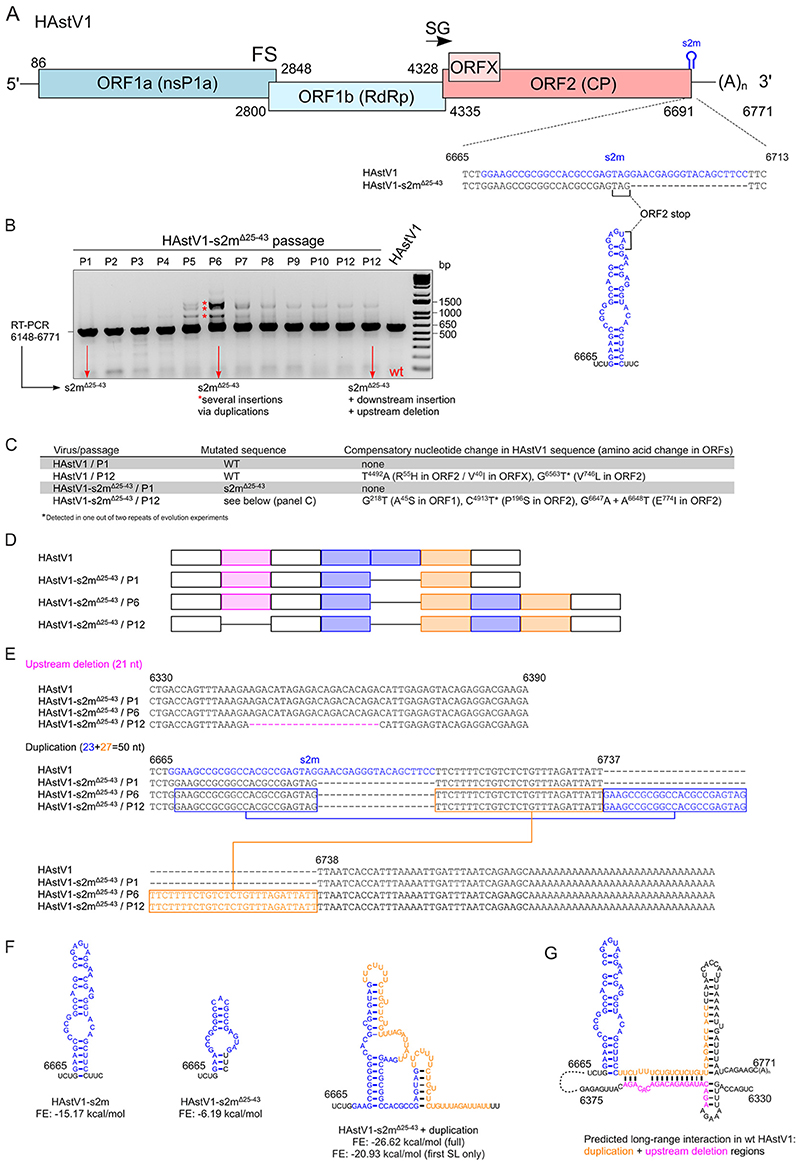
Evolution of HAstV1-s2m^Δ25-43^. (A) Schematic representation of the HAstV1 infectious clone genome used to generate s2m-deficient virus (HAstV1-s2m^Δ25-43^). HAstV1 genome elements: ORF, open reading frame; RdRp, RNA-dependent RNA polymerase; CP, capsid protein; FS, frameshift site; SG, site of subgenomic mRNA synthesis. (B) RT-PCR amplification of the s2m-flanking region of wild-type (WT) and evolving s2m-deficient HAstV1 genomes. (C) The results of whole-genome sequencing of passages 1 and 12 for HAstV1 and HAstV1-s2m^Δ25-43^ after serial passaging in Caco2 cells. (D) The schematics of detected rearrangements in the evolved HAstV1-s2m^Δ25-43^ (passages 1, 6, and 12). (E) The aligned and annotated sequences of s2m (in blue), upstream deletion (in pink), and downstream duplication (in blue for s2m-duplication, in orange for downstream duplication) regions in the WT and evolved HAstV1-s2m^Δ25-43^ (passages 6 and 12). The nucleotide positions are given for the WT HAstVI (pAVICI) sequence. (F) The structure of HAstV1 s2m, the HAstV1-s2m^Δ25-43^ s2m region, and evolved HAstV1-s2m^Δ25-43^ with duplication is shown. The secondary structure prediction was performed using the RNAfold WebServer based on ViennaRNA Package 2.0. FE, free energy predicted for each structure. (G) Predicted long-range RNA interaction between the downstream of s2m region and the sequence corresponding to the upstream deletion in the 12th passage of HAstV1-s2m^Δ25-43^.

The s2m element is present in diverse virus families that include astroviruses. Besides the s2m element, astroviruses and coronaviruses share a similar modular organization of their genomes, including the ordering of nonstructural and structural genes and a frameshift signal (Fig. 8A). The s2m sequence in human astrovirus 1 (HAstV1) spans the capsid-coding region and 3′ UTR, so the s2m-deficient virus, (HAstV1-s2m^Δ25–43^, Fig. 8A) was designed and rescued with 19 nt of the noncoding half of s2m deleted (44). Unlike the generated rIBVs with s2m modifications, HAstV1-s2m^Δ25–43^ exhibited a <log reduction in replication *in vitro* (44).

To establish whether deletion of s2m can also result in adaptive changes in the astrovirus genome, HAstV1-s2m^Δ25–43^ was serially passaged in Caco2 cells. Multiple changes in s2m and surrounding regions were observed, first noticeable at passage 5 (Fig. 8B). When sequencing the 6th and the 12th passage of HAstV1-s2m^Δ25-43^, an insertion consisting of a duplication of the remaining s2m and downstream sequence from the 3′ region preceding the poly-A tail was identified. Unlike the 36-nt insertion observed for IBV, which was 5 nt smaller than the deleted s2m sequence, this insertion consisted of 50 nt, 31 longer than the deleted s2m sequence (Fig. 8E). Interestingly, the 12th passage of HAstV1-s2m^Δ25–43^ acquired an in-frame deletion of 21 nt upstream of the s2m at positions 6346 to 6366 (Fig. 8C to E). Not only did this deletion result in the in-frame truncation of the C-terminal part of the capsid polyprotein, previously demonstrated to be dispensable in cell culture for the MLB group of astroviruses (45), but it also meant that, overall, P12 of HAstV1-s2mΔ^25–43^ only contained 10 nt of additional sequence length.

The evolutionary strategy observed for HAstV1 s2m^Δ25–43^ therefore echoes the results obtained for BeauR-dels2m (Fig. 2), suggesting the importance of sequence length rather than exact nucleotides of the s2m in both viruses. This recombination-prone region in both viruses also indicates the involvement of similar RNA-RNA interactions during viral replication that were utilized to restore sequence length and which may or may not have restored s2m-related function. Interestingly, in contrast to BeauR-dels2m-36-INS secondary RNA structure prediction (Fig. 3 and 4), the duplicated region in HAstV1-s2m^Δ25-43^ was predicted to regenerate the stem-loop structure (Fig. 8F). Furthermore, the deletion observed in the 12th passage was predicted to interact with the duplicated region (Fig. 8G).

## Discussion

Since the outbreak of SARS-CoV-2, interest in the coronavirus s2m genetic element has increased due to its potential as an antiviral drug target (18, 46, 47), which was previously considered for SARS-CoV (26). The significance and biological role of the s2m stem-loop in coronavirus replication remains to be determined. A recent study of SARS-CoV-2 identified that the s2m stem-loop interacts with host microRNA (miRNA) 1307-3p as well as the nucleocapsid (N) protein (48). In this study we investigated whether the s2m stem-loop is required for replication of the *Gammacoronavirus* IBV, the first coronavirus in which this sequence and associated predicted structure was identified (22). Investigation of IBV strains from different serotypes and genotypes (Fig. 1A) suggests the nucleotide sequence of the IBV s2m is largely conserved, with only a few nucleotide differences. The presence of the s2m sequence in multiple IBV strains would suggest it may not be a dispensable sequence and is potentially required for viral replication.

This study, however, suggests that the s2m stem-loop is not essential for IBV replication, as rIBVs with either the canonical stem-loop structure disrupted or the nucleotides encoding the s2m deleted had comparable growth kinetics *in vitro* to the parental Beau-R (Fig. 6). Despite the observations that modifications to the s2m sequence in the genome of the human astrovirus resulted in reduced replication *in vitro* (44), our findings that modifications to the IBV s2m sequence did not affect replication are not entirely unexpected, as not all coronaviruses contain an s2m-associated sequence or -associated RNA structure (24). A previous study investigating sequences deposited in GenBank did not identify an s2m-associated sequence in any of the available sequences corresponding to alphacoronaviruses (24) or the betacoronaviruses, apart from SARS-CoV. An s2m sequence and associated structure have been identified in SARS-CoV-2, with a couple of nucleotide differences to that of SARS-CoV (18). Although SARS-CoV-2 isolates have been identified with deletions in the s2m sequence (40, 49, 50), questions remain as to whether such deletions result in a fitness cost (23, 50). Using reverse genetics for deletion of the s2m sequence from SARS-CoV-2, no differences in viral replication *in vitro* or *in vivo* were identified (44). The *in vivo* studies used the Syrian hamster model for SARS-CoV-2, so it remains possible that the s2m stem-loop may play a role following infection of humans. Interestingly, in our study using *ex vivo* TOCs, altered growth kinetics were observed (Fig. 7), suggesting that the IBV canonical s2m stem-loop structure may be advantageous for replication in tracheal epithelial cells, the site of IBV replication during *in vivo* infection. This may suggest a role of the s2m stem-loop during *in vivo* infection that cannot be readily observed during *in vitro* assays.

Characterization of rIBV BeauR-dels2m, which had the nucleotides that constitute the s2m structure deleted, identified a 36-nt insertion in place of the deleted s2m sequence. NGS analysis suggested that this insertion was present as a minority population in the stock virus produced following rescue of the rIBV that became dominant after one passage *in vitro* in three different cell types (Fig. 2, Table 1). The insertion did not contain nucleotides that constitute the s2m sequence but, rather, consisted of the duplication of flanking sequences of the deleted s2m sequence (Fig. 2B). Interestingly, the downstream boundary of this duplication corresponds to the octanucleotide motif, GGAAGAGC, a sequence conserved among many coronaviruses (51). A similar result occurred during passaging of s2m-deficient HAstV1 (Fig. 8). Unlike the 36-nt insertion observed in BeauR-dels2m, the s2m-like stem-loop structure was predicted to be partially restored, and another upstream region was deleted, suggesting the location of important RNA elements that orchestrate virus replication. Interestingly, another s2m-containing astrovirus, VA1, was not viable upon deletion of s2m, whereas replication of SARS-CoV-2 was not affected by s2m deletion (44), suggesting a virus-specific function of this region.

The 3′ UTR of IBV, similar to all members of the coronavirus family, contains several secondary and tertiary RNA structures (9, 17, 18), including a stem-loop (11, 15) and a pseudoknot (13, 19, 20) in addition to the s2m stem-loop (22). It is possible that the deletion of the nucleotides encoding the s2m sequence may have negatively impacted the other RNA structures present as well as the overall structure of the 3′ UTR, including the neighboring ORF 7 (Fig. 3, 4). The models of secondary RNA structures, although informative, represent for the structures presented, the lowest free-energy states that the specific nucleotide sequences can assemble into. The models used do not take into consideration that the 3′ UTR is dynamic and interacts with other parts of the genome, including the 5′ UTR (17), or that interactions between both positive- and negative-sense RNAs occur during the coronavirus replication cycle. Advances in the ability to map RNA structure by selective 2' hydroxyl acylation analyses by primer extension (SHAPE) and dimethyl sulfate (DMS)-mutational profiling with sequencing (MaPseq) have provided a wealth of information regarding the coronavirus genome, specifically that of SARS-CoV-2 (29, 52, 53). In live cells the model of the SARS-CoV-2 s2m stem-loop deviates from the crystal structure of the SARS-CoV s2m (18), with the authors suggesting that these differences may be the result of the effect of *in vivo* interactions not being captured during *in silico* analysis. It is therefore possible that the 36-nt insertion introduces RNA structures more extensive than the model indicates, and likewise it is also possible that the impact of the deletion of the s2m sequence is more severe than presented in the RNA structure models.

In summary, the data in this study identify that the canonical s2m hairpin stem-loop RNA structure is not required for IBV replication, but it may enhance replication in tracheal tissue, the site of IBV infection *in vivo*. The difficulty in recovering a ″clean″ isolate of rIBV BeauR-dels2m alongside the *in vitro* passaging and NGS data which identified a 36-nt insertion in place of the deleted sequence, indicates there is a preference for nucleotide occupation in the s2m genome location, a finding mirror by insertions identified in a s2m deletion mutant of HAstV1. This preference for nucleotide occupation is independent of the specific s2m sequence, which may suggest similar functionality of s2m in both viruses and suggests that the length of the s2m-containing part of the genome is important for viral replication.

## Materials And Methods

### Ethics statement

Primary cells and organ cultures were prepared from chickens and chicken embryos in accordance with the Home Office guidelines of the United Kingdom (UK) and the UK Animals (Scientific Procedures) Act 1986. Animals were raised and all procedures performed in licensed facilities at The Pirbright Institute (TPI;X24684464). The use of animals for the preparation of cells and organ cultures was approved by the local animal welfare and ethical review committee. Embryonated hens' eggs were provided by The National Avian Research Facility (The Roslin Institute).

### Cells and viruses

Primary chicken kidney (CK) cells were generated by trypsinization of kidneys harvested from 2- to 3-week-old specific-pathogen-free (SPF) Rhode Island red (RIR) chickens by the Central Services Unit at The Pirbright Institute as previously described (54). Vero and DF1 cells (55) were obtained from the cell culture bank at The Pirbright Institute and maintained in Eagle's minimal essential medium (EMEM) and Dulbecco's modified Eagle's medium (DMEM) supplemented with 10% fetal bovine serum (FBS) and 1 mM L-glutamine, respectively. Caco2 cells were maintained in DMEM supplemented with 10% FBS, 1 mM L-glutamine, antibiotics, and nonessential amino acids. All cells were maintained at 37°C and confirmed negative for mycoplasma upon testing.

All isolates of IBV and rIBVs were propagated in 10-day-old SPF embryonated hens′ eggs. Eggs were supplied by VALO Biomedia, Germany. Allantoic fluid was harvested 24 to 48 h postinfection (hpi), clarified by low-speed centrifugation, and quantified by titration on CK cells. rIBV Beau-R is a molecular clone of Beau-CK (NCBI accession number AJ311317) and has been described previously (30). All IBV nucleotide positions mentioned are in relation to this reference sequence.

### Generation of recombinant IBV (rIBV)

The methods used to generate rIBV using a vaccinia virus-based reverse genetic system have been described previously (30, 31, 56). Briefly, a cDNA copy of the IBV genome was assembled within the genome of a recombinant vaccinia virus, and transient dominant selection, a method that takes advantage of recombination events between homologous sequences, was used to modify this IBV cDNA. Once the desired modification (detailed below) to the IBV cDNA was made correctly, as confirmed by Sanger sequencing, the rescue of infectious rIBV was carried out in CK cells. All nucleotide positions noted are in relation to the Beau-CK sequence (accession number AJ311317), with the nucleotide sequence, AGTGCCGGGGCCACGCGGAGTACGATCGAGGGTACAGCACT, of the s2m hairpin loop occupying positions 27472 to 27512. Briefly, nucleotide positions 27472 to 27512 were deleted from Beau-R, generating rIBV BeauR-dels2m, and the nucleotides GCC at positions 27481 to 27483 were deleted from Beau-R to generate BeauR-delGCC. Stock viruses were generated after three passages in CK cells and one passage in embryonated hens' eggs.

### Determination of the genomic 5′ end sequence

RNA extracted from the stock of Beau-R, BeauR-delGCC, and BeauR-dels2m, as well as passaged isolates of BeauR-dels2m, was reverse transcribed using Superscript IV reverse transcriptase (Invitrogen) following the manufacturer's protocol alongside the random oligonucleotide 5′-GTTTCCCAGTCACGATCNNNNNNNNNNNNNNN-3′. IBV-specific oligonucleotides 5′-GGCT GGTTCGAGTGCGAG-3′ and 5′-TGCAATACGCTGTGGTAATTC-3′ were used in a PCR using recombinant *Taq* polymerase to amplify nucleotides 264 to 1161 of the 5′ UTR and nonstructural protein (NSP) 2, with the resulting PCR product being Sanger sequenced. The remaining sequence of the 5′ UTR was determined using a 5′ RACE system for rapid amplification of cDNA ends (Invitrogen) using IBV-specific oligonucleotide 5′-TGTCTGCTCACTAAAC-3′ for the reverse transcription step and 5′-AGAACGTAGCCCAACGC-3′ for the amplification of dC-tailed cDNA step. Note that the dTd tailing reaction was performed on ice. The resulting PCR products were Sanger sequenced.

### Assessment of replication *in vitro*

CK, Vero, or DF1 cells seeded in 6-well plates were inoculated with 10^4^ (multiplicity of infection [MOI], ∼0.001) PFU of either rIBV Beau-R, BeauR-del-s2m, or BeauR-delGCC and incubated for 1 h, after which the inoculum was removed, and the cells were washed twice with phosphate-buffered saline a (PBSa). Per well, 3 mL serum-free BES ([N, N-Bis(2-hydroxyethyl)-2ami-noethanesulphonic acid]) medium (31) was added. Supernatant was harvested at 24-h intervals and assessed for infectious viral progeny via titration in CK cells.

### Assessment of plaque morphology and plaque size

Measurements of individual plaque sizes in CK cells were taken using ImageJ as previously described (57). Per virus, a total of 30 individual plaques from two independent wells were counted.

### Assessment of ciliary activity in *ex vivo* tracheal organ cultures (TOCs)

TOCs were prepared from 19-day-old SPF Valo embryonated hens' eggs as previously described (58). Briefly, the trachea was removed from each embryo and sectioned using a microtome to generate rings of approximately 1 mm; one ring (otherwise termed TOC) was seeded per tube. Each TOC was inoculated with 10^4^ PFU of either rIBV Beau-R, BeauR-delGCC, or BeauR-del-s2m, in 500 *μ*L of TOC medium (0.5 × EMEM, 75 *μ*M *α*-methyl-D-glycoside, 40 *μ*M HEPES, 0.1% sodium bicarbonate, 10 U/mL penicillin, 10 *μ*g/mL streptomycin) or mock infected with 500 *μ*L TOC medium in replicates of 10 and incubated at 37°C, with 7 to 8 revolutions per h. The percentage of ciliary activity of each TOC was assessed at 24-h intervals using a light microscope as previously described (42, 59).

### Assessment of viral replication in *ex vivo* TOCs

TOCs were prepared from 2-week-old SPF RIR chickens as previously described (58). Briefly, the trachea was removed from each chicken and cleaned using a scalpel blade. The tracheas were flushed with PBS prior to sectioning using the microtone. Two tracheal rings (TOCs), of approximately 2 to 3 mm, were seeded per tube, with this counting as one replicate. Per experiment, each replicate was inoculated with 10^4^ PFU of either Beau-R, BeauR-delGCC, or BeauR-dels2m and incubated for 1 h at 37°C. After 1 h the inoculum was removed and the TOCs were washed twice with PBSa to remove any unbound virions, after which 1 mL TOC medium was added. The infected TOCs were incubated at 37°C, with 7 to 8 revolutions per h. Supernatant was harvested at 24-h intervals and assessed for infectious viral progeny via titration in CK cells.

### Serial passage of rIBV in *vitro*

For the first passage, confluent CK, Vero, or DF1 cells in six-well plates were washed with PBSa and inoculated in triplicate with 10^4^ PFU of either rIBV Beau-R, BeauR-dels2m, or BeauR-delGCC or BES medium for mock infection and incubated 1 h. Following attachment, cells were washed twice with PBSa to remove residual virus, after which 3 mL BES medium was added per well. Extracellular virus was harvested when cells displayed extensive IBV-induced cytopathic effect (CPE): 24 hpi for CK cells, 72 hpi for Vero and DF1 cells. For the remaining passages, supernatant was diluted in BES medium either 1/100 for CK cell infection or 1/10 for Vero cell infection or used neat for DF1 cell infection; incubation times remained the same as for passage 1. The presence of rIBV in the supernatant was confirmed by reverse transcriptase PCR (RT-PCR) analysis using a random oligonucleotide for the RT step and IBV-specific oligonucleotides targeting the 3′ UTR as described previously (60). The resulting PCR products were Sanger sequenced to identify nucleotide changes in the 3′ UTR.

### Modeling of RNA secondary structure

Secondary RNA structure modeling was performed using RNAfold WebServer (*http://rna.tbi.univie.ac.at/cgi-bin/RNAWebSuite/RNAfold.cgi*) based on ViennaRNA Package 2.0, selecting the minimum free energy (MFE) model option. Nucleotides from positions 27334 to 27635 or 27103 to 27635 of reference sequence AJ311317, encompassing a total of 302 or 533 nucleotides, were modeled.

### Next-generation sequencing

RNA was prepared from 2 mL of stock virus of BeauR-dels2m (otherwise termed BeauR-dels2m-36nt-INS) and a passaged isolate (passage 1 in CK cells) of BeauR-dels2m-36nt-INS following a previously published protocol (61). The sequencing data generated were assembled using an inhouse pipeline. The pipeline trimmed reads with Trim Galore as a quality control step and assembled reads in the SPADes assembler iteratively by subsampling the reads by their k-mer content. The contigs produced at each iteration were considered trusted contigs, and the final assembly was scaffolded using reference genome AJ311317. The reads were also aligned using the BWA aligner, and the pileup of this alignment was used to call variants with the low-frequency variant called SiNPle (62).

### Serial passage of recombinant HAstV1 *in vitro*

The recombinant HAstV1 and HAstV1-s2m^Δ25-43^ virus stocks were passaged in duplicates in Caco2 cells using a multiplicity of infection of 0.1 as previously described (44, 63). Briefly, Caco2 cells were infected at an MOI of 0.1 and incubated for 96 to 120 h until the appearance of CPE, freeze-thawed twice, filtered through a 0.2-*μ*m filter, and supplemented with 5% glycerol. Virus RNA was isolated with a Direct-zol RNA MicroPrep kit (Zymo Research). The sequence of the s2m was analyzed by RT-PCR and Sanger sequencing (44).

### Statistics

All statistical analyses were performed using GraphPad Prism version 8.0. Normality and the standard deviation of each data set were assessed prior to each statistical test.

## Figures and Tables

**Table 1:** Estimated allele frequency of the variants in BeauR-dels2m and BeauR-dels2m-P1 CK^a^

Position	Variation	BeauR-dels2m	BeauR-dels2m-P1-CK
10464	GtoT	Yes (0.99)	Yes (1)
27458	G to A	Yes (0.90)	Yes (0.99)
27474	T to G	Yes (0.94)	Yes (1)
27512	T to C	No	Yes (0.4)
27465	36-nt insertion	Yes (0.51)	Yes (0.78)

a Variants were identified using freebayes. The allele frequencies (in parentheses) for the small variants were estimated using the formula allele frequency = AO/(AO + RO), where AO is the number ofreads with an allele reported byfreebayes, and RO is the number of reads with reference reported by freebayes.The sequence used to detect the insertion is as follows: GTATAAAGATGCCAGGACGCCCATTAGGGGAAGAGC.

## Data Availability

The sequencing data have been submitted to the sequence read archive (SRA) with reference PRJNA929486.

## Importance

Coronaviruses infect many species, including humans and animals, with substantial effects on livestock, particularly with respect to poultry. The coronavirus RNA genome consists of structural elements involved in viral replication whose roles are poorly understood. We investigated the requirement of the RNA structural element s2m in the replication of the *Gammacoronavirus* infectious bronchitis virus, an economically important viral pathogen of poultry. Using reverse genetics to generate recombinant IBVs with either a disrupted or deleted s2m, we showed that the s2m is not required for viral replication in cell culture; however, replication is decreased in tracheal tissue, suggesting a role for the s2m in the natural host. Passaging of these viruses as well as human astrovirus lacking the s2m sequence demonstrated a preference for nucleotide occupation, independent of the s2m sequence. RNA modeling suggested deletion of the s2m may negatively impact other essential RNA structures.
